# Towards Data-Driven Vehicle Estimation for Signalised Intersections in a Partially Connected Environment

**DOI:** 10.3390/s21248477

**Published:** 2021-12-19

**Authors:** Roozbeh Mohammadi, Claudio Roncoli

**Affiliations:** Department of Built Environment, School of Engineering, Aalto University, 02150 Espoo, Finland; claudio.roncoli@aalto.fi

**Keywords:** traffic state estimation, connected vehicles, data-driven estimation

## Abstract

Connected vehicles (CVs) have the potential to collect and share information that, if appropriately processed, can be employed for advanced traffic control strategies, rendering infrastructure-based sensing obsolete. However, before we reach a fully connected environment, where all vehicles are CVs, we have to deal with the challenge of incomplete data. In this paper, we develop data-driven methods for the estimation of vehicles approaching a signalised intersection, based on the availability of partial information stemming from an unknown penetration rate of CVs. In particular, we build machine learning models with the aim of capturing the nonlinear relations between the inputs (CV data) and the output (number of non-connected vehicles), which are characterised by highly complex interactions and may be affected by a large number of factors. We show that, in order to train these models, we may use data that can be easily collected with modern technologies. Moreover, we demonstrate that, if the available real data is not deemed sufficient, training can be performed using synthetic data, produced via microscopic simulations calibrated with real data, without a significant loss of performance. Numerical experiments, where the estimation methods are tested using real vehicle data simulating the presence of various penetration rates of CVs, show very good performance of the estimators, making them promising candidates for applications in the near future.

## 1. Introduction

Urban traffic congestion is a widespread phenomenon appearing in most cities worldwide, bringing a range of negative impacts on the quality of citizens’ lives and environment. A sustainable way of dealing with urban traffic congestion is the implementation of advanced traffic control strategies that aim at improving the utilisation of the existing infrastructure [1]. In particular, various adaptive traffic control strategies have been proposed during the last decades to facilitate traffic movement in urban signalised intersections, including, e.g., [2,3,4,5,6,7,8].

The availability of accurate and reliable real-time information is a prerequisite for running efficient adaptive signal control strategies. For this purpose, various infrastructure-based sensors, such as, e.g., loop detectors, radars, cameras, and magnetometers, have been employed to collect the necessary measurements [9]. However, these data collection tools have several deficiencies. The installation and maintenance cost of these devices are considerably high [10], considering that for many adaptive strategies, at least one device is needed for each approach of a signalised intersection. In addition, some of these sensors, such as loop detectors, are so-called point detectors, i.e., they are able to detect a vehicle only when it is located at a specific position (typically, when a vehicle is exactly above the detector), while no information is collected for any location upstream or downstream of the detector position. On the other hand, other types of sensors, such as cameras and radars, are capable of recording vehicle movements over some, still limited, space; however, their performance may be affected by various exogenous factors, including adverse weather conditions, improper lighting, or signal interference [11,12]. In order to deal with incomplete measurements retrieved from infrastructure-based sensors, various studies proposed the usage of estimation techniques, aimed at supplementing missing data (see, e.g., [13,14,15]).

Recent years have seen a rapid development of Connected Vehicle (CV) technology, introducing opportunities for collecting a large amount of real-time accurate vehicle data. Reliance on CV data may replace the need of infrastructure-based sensors for advanced traffic control, allowing also to increase the granularity of data at a reduced cost. Existing technologies already enable CVs to collect detailed trajectory data based on positioning systems, such as, e.g., GPS, GLONASS, Galileo, and COMPASS, and to transfer them via, e.g., dedicated short-range communications (DSRC) or mobile phone network, including the recently developed 5G [16]. To account for such data availability, various applications for traffic signal control exploiting CV data have been already envisioned and developed [17]. However, it is expected that a 100% penetration rate of CVs, which would allow a perfect traffic detection without additional sensors, is not going to be reached in the near future. In fact, according to [16], it is reasonable to expect a penetration rate of CVs between 40% and 62% by 2030. Consequently, there is a need to develop novel methods to compensate for the incomplete availability of CV data during this transition period, before we reach a fully CV environment.

Several estimation methods are being developed in order to compensate for the limited number of CVs (note that some of the cited papers utilise the term “probe vehicle” instead of “connected vehicle”; as in this paper we do not deal with automation, we consider these two terms as interchangeable. Therefore, for the sake of consistency, we are using the term CV throughout the entire paper.) during the transition period before reaching a fully connected environment. On the one hand, being the required input for many traffic signal control methods, queue length estimation has been the focus of several previous works. As an example, ref. [18] proposed a queue length estimation method by integrating CV data with shockwave theory, using data mining techniques; the robustness of the method was tested on the NGSIM data, showing promising accuracy in queue length estimation. The recent study [19] developed a cycle-based queue length estimation by fusing historical and real-time data from CVs, using a maximum likelihood estimation method. Other studies in this area include [20,21,22,23,24]. Another stream of works dealt with the estimation of total vehicle counts, which include both queuing and moving vehicles. The issue of incomplete CV data availability is typically addressed by applying data fusion techniques that integrate infrastructure-based sensor data and CV data. For instance, ref. [25] proposed a method to fuse traffic camera data and CV data in order to estimate traffic state in urban streets; while [26] employed a data fusion method considering CVs and loop detectors, where in order to solve the problem of low CV penetration rate, a probability-based approach is applied to estimate the position of the queue tail. Similar data fusion approaches have been employed in other studies, including, e.g., [27,28]. More recently, in [29] a traffic volume estimation method was proposed by assuming a time-dependent Poisson process with a constant arrival flow rate of vehicles, while the authors of [30] estimated queue length and traffic volume by applying probability theory on CV data. Furthermore, a Kalman filter-based method was developed in [31] for vehicle count estimation at signalised intersections, relying only on CV data; the method is applied for a system where the traffic flow conservation equation is used as state equation, while the measurement equation is defined based on hydrodynamic relations of traffic flow. Moreover, a multi-lane vehicle estimation method have been proposed in [32].

Despite the novelty and effectiveness of the aforementioned methods, there are some limitations that may prevent their usage in practice. First, methods delivering only queue estimation may not provide sufficient inputs for some signal control strategies, such as signal timing methods that require an estimate of the arrival time for each individual vehicle, including, e.g., [8,33]; as well as strategies that require total vehicle densities or flows such as SCATS and SCOOT [34]. Furthermore, to our best knowledge, all model-driven methods aimed at estimating total vehicle counts, i.e., both queuing and moving vehicles, require, in addition to CV data, at least an infrastructure-based point detector that provides vehicle arrival flow rate or employ strict assumptions on vehicles arrival rates.

In contrast, data-driven methods have gained recent popularity thanks to their ability to allow identifying complex patterns and correlations by learning them from available data [35,36,37,38,39]. Nevertheless, data-driven models typically need a large amount of data used for training, which may not be easy and inexpensive to collect [40]. To the best of our knowledge, the only data-driven method proposed for traffic density estimation of urban signalised links based on CV data is [41], where the authors develop a method based on artificial neural networks (ANN), random forest (RF), and k-nearest neighbors algorithm (K-NN) for traffic density estimation employing CV data. The proposed method was trained and tested on synthetic data, produced via microscopic simulation.

We present in Table 1 a collection of the most relevant research works on traffic estimation for urban signalised links using CV data. In summary, previous studies mainly focused on estimating total vehicle counts or queue lengths by fusing data from multiple sources, such as CV data and point detectors. Fewer studies utilised only CV data, where the proposed methods are based on various mathematical models derived from, e.g., traffic flow theory and probability theory; to deal with data incompleteness, such methods require more or less strict assumptions, e.g., on the arrival flow rate, the arrival patterns of CVs, or their penetration rate. However, these methods are only capable to estimate queue length at lane level or number of vehicles at link level. Moreover, there are no existing methods that are providing estimates with a higher spatial granularity, e.g., intra-lane vehicle counts.

In this paper, we propose two data-driven estimation methods based on machine learning models, to estimate the number of vehicles approaching a signalised intersection by employing only information collected from a limited amount of CVs, which can be utilised, e.g., for operating adaptive signal timings. A first method, denoted as “aggregated”, is designed to provide estimates for the total number of vehicles present in a signalised link; whereas the second method, denoted as “disaggregated”, delivers a more granular estimation, being designed to estimate the number of non-connected vehicles upstream and downstream of each CV. By applying the two developed estimation methods, not only the total vehicle count can be estimated, but also the number of vehicles between each pair of CVs, which leads to a higher resolution of vehicle estimation with respect to existing approaches. Moreover, we present a novel method to address the need for large amount of data to train the estimation models, based on limited amount of real data collected from the field. In this procedure, a relatively small real data can be collected offline, e.g., from a fixed camera or an unmanned aerial vehicle (i.e., drone). Then, a microscopic traffic simulation is employed to expand the training data without compromising the estimation accuracy. In addition, we investigate the sensitivity of the estimation performance with respect to the amount of data utilised for model training. Finally, we also explore estimation performance under different percentages of connected vehicles.

The remainder of this paper is organised as follows. Section 2 describes the proposed estimation methods. In Section 3, we describe the data utilised in this paper, as well as its processing. Model training results are presented in Section 4, while estimation performance with real data are included in Section 5. Lastly, Section 6 presents a summary and discussion of key findings, while also outlining future research directions.

## 2. Data-Driven Vehicle Estimation

### 2.1. Estimation Framework

**Figure 1:**
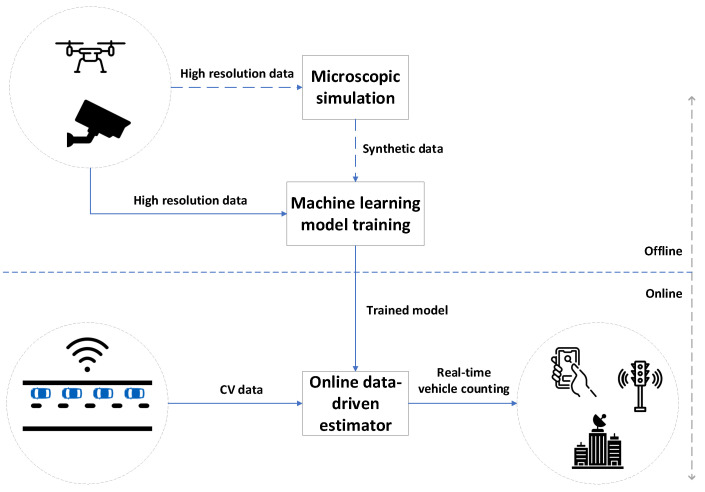
Illustration of the proposed estimation methodology. Note that employing microscopic simulation data is not strictly necessary and it may be used for model training if the high-resolution real data is deemed insufficient.

The objective of our proposed data-driven methodology is to estimate the number of vehicles on a road segment approaching a signalised intersection by employing only CV data in mixed traffic conditions. The proposed method consists of a set of models that require to be trained offline using high resolution data, such as vehicle trajectories that can be collected, e.g., by cameras installed (temporarily) on the infrastructure or from a drone. As the models may require large amount of data, a cost effective approach in case the available data is not deemed sufficient is to collect a limited amount of data and use it for calibrating a microscopic simulation tool, which can be then used to produce virtually unlimited amount of synthetic data for model training. However, we show later in the paper (in Section 4.2) that 1 h of data is actually sufficient to achieve an acceptable accuracy level. Finally, the online implementation consists in feeding the trained models with CV data collected in real-time to estimate vehicle counting, which can be then transmitted to the signal controller or other users. The whole process is illustrated in Figure 1.

In particular, we propose two methods for estimation. The first method directly estimates the total number of vehicles by using aggregated information retrieved from all the CVs travelling along a segment. The second method is developed in a disaggregated fashion, utilising information from pairs of consecutive CVs travelling along the segment, as well as from individual CVs. The latter approach allows a more granular estimation, which may be useful for a future generation of traffic signal control systems, designed to operate with high resolution vehicle data, including, e.g., [8,33,45]. In the following sections, we elaborate first on the assumptions and formulation related to each estimator; we then continue by presenting the common framework utilised for building the machine learning models; and finally we present the related data settings and training procedures.

### 2.2. Aggregated Estimation Method

Here, we elaborate on a method to estimate the total number of vehicles present in a signalised link, based on aggregated data collected from CVs. We assume that data from CVs is available within a reasonable distance from the intersection, e.g., in a range of 200 m–1 km, which we denote as the detection area, defined dependent on the infrastructure characteristics. Moreover, we assume that CV data is available in real-time, i.e., with negligible communication delays; this could be reasonably achieved by employing various existing communication technologies [46], which are therefore not explored in this work.

Let us introduce V(k) as the set of vehicles present within a segment at time *k* and S(k)⊂V(k) as the set of CVs present within a segment at time *k*. To develop the proposed model, we assume availability of the following variables for each CV i∈S(k):

- $d_{i}\left(k\right)$ as the distance between vehicle *i* and the stop-bar at time step *k*;
- $v_{i}\left(k\right)$ as the instantaneous speed of vehicle *i* at time step *k*;
- $t_{i}\left(k\right)$ as the time in the detection area of vehicle *i* at time step *k*, obtained as
  (1) $$t_{i}\left(k\right)=k-{\hat{k}}_{i},$$
  where ${\hat{k}}_{i}$ is the time when vehicle *i* entered the detection area.
- $u_{i}\left(k\right)$ as the mean speed in the detection area of vehicle *i* at time *k*, obtained as
  (2) $$u_{i}\left(k\right)=\frac{L-d_{i}\left(k\right)}{t_{i}\left(k\right)},$$
  where *L* is the length of the detection area.

We now proceed by introducing the following notation for aggregated variables

(3) $$\begin{array}{cc} \omega^{\mathrm{avg}}\left(k\right) & =\frac{1}{\left|S(k)\right|}\sum_{i\in S(k)}\omega_{i}\left(k\right) \end{array}$$

(4) $$\begin{array}{cc} \omega^{\min}\left(k\right) & =\min_{i\in S(k)}\omega_{i}\left(k\right) \end{array}$$

(5) $$\begin{array}{cc} \omega^{\max}\left(k\right) & =\max_{i\in S(k)}\omega_{i}\left(k\right), \end{array}$$

where $\omega^{\mathrm{avg}}\left(k\right)$, $\omega^{\min}\left(k\right)$, and $\omega^{\max}\left(k\right)$ are, respectively, the arithmetic average, minimum, and maximum of variable ω for all CVs detected at time *k*. Note that variable ω is introduced here, for the sake of readability, as a *free variable*, i.e., it is a placeholder that can be substituted by actual variables that are used in our problem formulation.

The overall model to be estimated reads (where time index *k* is omitted for the sake of readability)

(6) $$\tilde{N}=f(d^{\min},d^{\max},v^{\mathrm{avg}},v^{\min},v^{\max},t^{\mathrm{avg}},t^{\min},t^{\max},u^{\mathrm{avg}},u^{\min},u^{\max}),$$

where $\tilde{N}$ is the estimated number of non-connected vehicles (at each time *k*) and the arguments of the function are calculated according to (3)–(5). Consequently, the total number of vehicles $\hat{N}\left(k\right)$ is calculated as the sum of the estimated number of non-connected vehicles and the (measured) number of CVs as

(7) $$\hat{N}\left(k\right)=\tilde{N}\left(k\right)+\left|S\left(k\right)\right|.$$

Finally, the output of the aggregated estimator, which is derived from (7), may be sent to the infrastructure to be used for intersection control.

We introduce here also the percentage of CVs (pctCV), which is used for numerical evaluations in Section 5, calculated as

(8) $$\mathrm{pctCV}\left(k\right)=\frac{\left|S(k\right|}{\left|V(k)\right|}.$$

### 2.3. Disaggregated Estimation Method

#### 2.3.1. Model Formulation

In order to exploit the additional details that are available from CVs, we propose here an alternative method that allow to obtain more granular estimation results.

**Figure 2:**
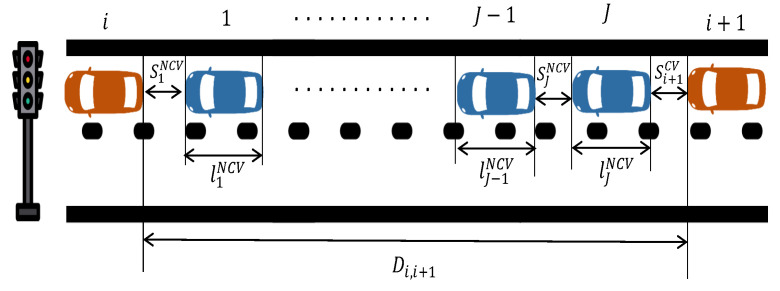
An example of vehicles approaching a signalised intersection; Red vehicles are CVs and blue vehicles are non-connected vehicles.

Let us consider a stream of vehicles travelling on a signalised segment, as shown in Figure 2, for which we assume availability of information for a pair of consecutive CVs, denoted as *i* and i+1. The front-bumper to rear-bumper distance between vehicle *i* and i+1 at a given time *k*, denoted by $D_{i,i+1}\left(k\right)$, can be calculated as

(9) $$D_{i,i+1}\left(k\right)=s_{i+1}^{\mathrm{CV}}\left(k\right)+\sum_{j=1}^{J}\left[s_{j}^{\mathrm{NCV}}\left(k\right)+l_{j}^{\mathrm{NCV}}\right],$$

where $s_{i}^{\mathrm{CV}}$ is the space-gap between CV *i* and its predecessor, $s_{j}^{\mathrm{NCV}}$ is the space-gap between non-connected vehicle *j* and its predecessor $l_{j}^{\mathrm{NCV}}$ is length of (non-connected) vehicle *j*, and $n_{i,i+1}$ is the number of (non-connected) vehicles between vehicle *i* and i+1. Introducing variables $\bar{s}$ and $\bar{l}$ as the average space-gap and vehicle length, respectively, for all vehicles (irrespectively if they are connected or not), allows us to approximate the number of vehicles between the *i*th and (i+1)th vehicles at time *k*, $n_{i,i+1}\left(k\right)$, as

(10) $$n_{i,i+1}\left(k\right)\approx \frac{D_{i,i+1}\left(k\right)-\bar{s}\left(k\right)}{\bar{l}+\bar{s}\left(k\right)}.$$

We know from existing literature that the behaviour of drivers and, in particular, their car-following behaviour, is affected by many variables, including vehicle relative positions, speeds, and accelerations, which have been investigated over the decades resulting in a variety of microscopic car-following models [47]. Moreover, the prevailing traffic conditions, road design, and other external factors, may affect such behaviour; see, e.g., [48,49]. However, estimating accurately the behaviour (and spacing) of each vehicle would be an extremely challenging task, due to the high number of parameters involved and the large amount of information that may be needed; see, e.g., [50,51,52].

On the other hand, by considering the fact that a vehicle is affected by the state of the preceding ones, our intuition is that, utilising a set of independent variables obtained for a pair of vehicles (e.g., CVs), as well as some mesoscopic variables characterising the overall traffic conditions, such as the mean speed of vehicles over a (short) time interval or a proxy for the delay, we may be capable of calculating the total number of vehicles in such area, i.e., implicitly estimating $\bar{s}$ and $\bar{l}$. Since formulating and solving such problem analytically would be extremely challenging, due to the non-linearities and high number of parameters, we employ again a data driven approach, where we train a set of machine learning models to estimate $n_{i,i+1}\left(k\right)$.

The assumptions on availability of CV information, as well as the notation for the measured variables, are the same as in Section 2.2; in addition, we assume availability of the instantaneous acceleration of vehicle *i* at time *k*, denoted by variable $a_{i}\left(k\right)$. Note that, in this context, one can view position, speed, and accelerations ($d_{i}\left(k\right)$, $v_{i}\left(k\right)$, and $a_{i}\left(k\right)$) as representative of a CV status, whereas $t_{i}\left(k\right)$ and $u_{i}\left(k\right)$ as proxy variables that reflect prevailing traffic conditions.

The overall model to be estimated reads (where time index *k* is omitted for the sake of readability)

(11) $$\begin{array}{cc} {\hat{n}}_{i,i+1}=f( & d_{i},d_{i+1},\Delta d_{i,i+1},v_{i},v_{i+1},\Delta v_{i,i+1},a_{i},a_{i+1},\Delta a_{i,i+1},t_{i},t_{i+1},\\ \Delta t_{i,i+1},u_{i},u_{i+1}), \end{array}$$

where ${\hat{n}}_{i,i+1}$ is the estimate for $n_{i,i+1}$ and $\Delta \omega_{i,i+1}$ denotes the relative value of variable ω, defined as $\Delta \omega_{i,i+1}=\omega_{i+1}-\omega_{i}$.

#### 2.3.2. CV Pair Clustering

Due to the effects of traffic signals, traffic characteristics at links leading to signalised intersections follow recurrent patterns, which feature distinct traffic phases in the vicinity of an intersection. Therefore, developing different models taking into account the different traffic phases is a potentially effective way to improve the estimation accuracy. A similar approach has been successfully applied in other research, such as, e.g., [53]. Accordingly, we assume that a signalised link is characterised by three traffic phases, namely, (a) queuing phase, (b) slowing-down phase, and (c) free-flow phase. In the queuing phase, vehicles are stopped or moving at a very low speed, typically due to a red traffic signal that generates a queue; in the slowing-down phase, vehicles are reducing the speed in order to stop at the stop-bar or to join the queue; while in the free-flow phase, vehicles are moving freely, at a speed close to their desired one. In order to account for these cases in our estimation, we implement clustering; namely, we associate each detected CV to a traffic phase, by comparing its speed with two pre-defined thresholds, where the first one ($\theta_{1}$) differentiates queuing vehicles from moving vehicles and the second one ($\theta_{2}$) differentiates slowing-down vehicles from free-flow vehicles.

Considering the above-mentioned three phases and their possible combinations, we build nine separate models for estimation of non-connected vehicles between a pair of CVs. We refer to each model by using two letters, one representative of the downstream vehicle phase and one representative of the upstream vehicle phase, where Q, S, and F, denote the queue, slowing-down, and free-flow phases, respectively. For example, the Q-S model is developed to estimate the number of non-connected vehicles between a pair of CVs when the downstream CV is in the queue phase and the upstream CV is in the slowing-down phase. Table 2 illustrates the criteria employed to cluster estimation models based on the speed of the downstream CV $v_{i}$ and the speed of the upstream CV $v_{i+1}$. Numerical comparisons will show the improvements that can be achieved by employing these models with respect to a unique model, implemented without applying any clustering.

#### 2.3.3. Estimating First and Last Vehicles

In order to estimate the total number of vehicles in a segment, in addition to the number of vehicles between each pairs of CVs, the following additional cases must be considered:

- when CV *i* is the closest to the stop-bar, then the number of non-connected vehicles between vehicle *i* and the stop-bar need to be estimated;
- when CV *i* is the farthest from the stop-bar, then the number of non-connected vehicles behind vehicle *i* need to be estimated;
- when there is only a CV *i* in the segment, then both the number of non-connected vehicles between vehicle *i* and the stop-bar as well as the number of non-connected vehicles behind vehicle *i* need to be estimated.

To address the first case, we develop an additional model to estimate the number of non-connected vehicles in front of CV *i*, by considering as input of the model only data from vehicle *i*. In this case, the model for estimation reads (where time index *k* is omitted for the sake of readability)

(12) $${\hat{n}}_{i}^{s}=f\left(d_{i},v_{i},a_{i},T_{i},u_{i}\right),$$

where ${\hat{n}}_{i}^{s}$ is the estimated number of non-connected vehicles between vehicle *i* and the stop-bar (at each time *k*).

Similarly as in the previous case, clustering based on different traffic phases may contribute to improve estimation results. Here, we develop three models, where the speed $v_{i}$ is employed to differentiate among phases, according to the rules shown in Table 3.

In order to address the second case, as the available information is scarcely instructive for estimating drivers’ behaviour, we consider a simpler, yet effective approach. That is, we place a dummy vehicle at the entrance of the detection area, where the speed of the dummy vehicle is equal to the free-flow speed, while acceleration and time in the detection area are set to zero. Consequently, the number of non-connected vehicles are estimated using the estimation method based on a pair of CVs presented earlier; we denote such estimate as ${\hat{n}}_{i}^{f}\left(k\right)$.

Finally, the third case is addressed by simply combining the methods proposed for the two previous cases.

#### 2.3.4. Estimating the Total Number of Vehicles via the Disaggregated Method

By combining the model for estimating the number of vehicles between each pair of CVs together with the methods for estimating the first and last vehicles, we are also able to estimate the total number of vehicles present in the segment at each time step as

(13) $$\hat{N}\left(k\right)=\sum_{i\in S(k)}{\hat{n}}_{i,i+1}\left(k\right)+{\hat{n}}_{\bar{i}}^{s}\;\left(k\right)+{\hat{n}}_{\tilde{i}}^{f}\;\left(k\right)+\left|S\left(k\right)\right|,$$

where $\bar{i}$ and $\tilde{i}$ are, respectively, the indexes for the first and last detected CVs, included in S(k).

### 2.4. Fully-Connected Feedforward Multi-Layer ANN

Various methods are available for building machine learning models, including decision tree [54], Bayesian network [55], and kernel machine [56]. In this work, we build our estimation models employing a fully-connected feed forward multi-layer ANN [57], which is characterised by the following advantages (see [58]). First, ANN is able to identify complex relations between a set of inputs and outputs for which analytical models do not exist or are difficult to define. Second, ANN can identify patterns of relationship between inputs and outputs even in the case of noisy data. Third, ANN can be applied to identify non-linear dependencies.

Typically, a fully-connected feedforward multi-layer ANN consists of three type of layers, namely an input layer, one or more hidden layers, and an output layer. Each independent variable that is used as input for the estimation is assigned to a neuron in the input layer. Then, a fully connected network of hidden layers connects the input layer to the output layer, where, in each layer, the output of a neuron is a function (called activation function) of the weighted sum of inputs. Considering this structure, the ANN model estimates the output variables using forward propagation as follows:

(14) $$o_{m}^{\left(l+1\right)}=f\left(w_{m}^{\left(l+1\right)}o^{\left(l\right)}+b_{m}^{\left(l+1\right)}\right),$$

where $o_{m}^{\left(l+1\right)}$ denotes the output from the *m*-th neuron in layer l+1, $o^{\left(l\right)}$ represents the output vector from the neurons in layer *l*, $w_{m}^{\left(l+1\right)}$ is a vector of weights between the *m*-th neuron in layer l+1 and all the neurons in layer *l*, $b_{m}^{\left(l+1\right)}$ denotes the bias term associated with the *m*-th neuron in layer l+1, and *f* is an activation function used to capture nonlinear relationships. In contrast with a conventional neural network that has only one hidden layer, a multi-layer ANN consists of several hidden layers that improve the efficiency in finding complex relationships between the input and the outputs variables. Nevertheless, increasing the number of hidden layers may cause overfitting to the training data, that is, a model is trained based on recognised patterns that are present in the training dataset, while such patterns are imperceptible in other datasets. In order to prevent overfitting, we may account for some or all the following countermeasures. First, a portion of the training dataset should be randomly extracted and treated as a validation dataset, which is used to compare the model performance on data that is not included in the training dataset [59]. Second, one may add two regularisation parameters, which are known as $L_{1}$ and $L_{2}$ in machine learning literature [60]. Third, one may add dropout layers, which can effectively prevent overfitting by randomly removing neurons; namely, each update of a layer during training is performed with a different unique neurons configurations, which in turn, reduces the possibility of overfitting [61].

**Figure 3:**
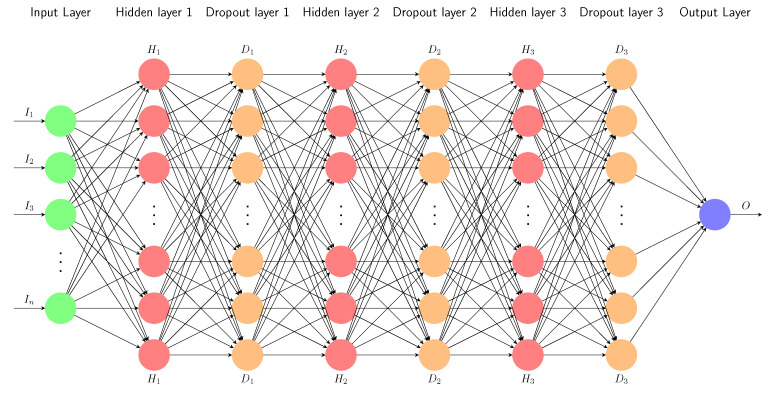
Structure of applied multi-layer ANN.

Considering the trade-offs in ANN modelling, we employ a multi-layer ANN that consists of an input layer, three hidden layers, three dropout layer, and an output layer. The number of neurons for each hidden layer is 64. Figure 3 presents a sketch of the structure of the chosen ANN that is used to build our estimation models. In addition, a Rectified Linear Unit (ReLU) [62] is used as activation function in the proposed multi layer ANN. ReLU activation function is a piece-wise linear function that outputs its input if it is positive and zero otherwise. In particular, ReLU allows a model to be quickly and properly trained by mitigating problem of vanishing gradient [63]. Finally, we train the estimation models using the Adaptive Moment Estimation (ADAM) optimiser, which is a computationally efficient algorithm suitable for noisy and sparse gradients, which also allows to easily tune the model hyperparameters compared to other methods [64].

### 2.5. Performance Metrics

In order to evaluate the trained models, we consider four performance metrics: the root-mean-square error (RMSE) and and mean absolute error (MAE), defined as in [65], and the normalised root mean squared error (NRMSE) and normalised mean absolute error (NMAE), defined as in [35,66], which are formulated as

(15) $$\begin{array}{cc} \mathrm{RMSE} & =\sqrt{\frac{\sum_{i=1}^{n}{\left(y_{i}-\hat{y_{i}}\right)}^{2}}{n}} \end{array}$$

(16) $$\begin{array}{cc} \mathrm{MAE} & =\frac{\sum_{i=1}^{n}\left|y_{i}-\hat{y_{i}}\right|}{n} \end{array}$$

(17) $$\begin{array}{cc} \mathrm{NRMSE} & =\frac{\sqrt{n\sum_{i=1}^{n}{\left(y_{i}-\hat{y_{i}}\right)}^{2}}}{\sum_{i=1}^{n}y_{i}} \end{array}$$

(18) $$\begin{array}{cc} \mathrm{NMAE} & =\frac{\sum_{i=1}^{n}\left|y_{i}-\hat{y_{i}}\right|}{\sum_{i=1}^{n}y_{i}}, \end{array}$$

where $y_{i}$ and $\hat{y_{i}}$ denote, respectively, the observed and estimated values of *i*-th sample and *n* is number of samples in the dataset. The RMSE penalizes variance as it gives more weight to errors with larger absolute values while MAE consider identical weight for all errors [67]. However, RMSE and MAE do not consider the scale of actual estimation, which may cause an improper comparison, particularly when the scales are different. In this regard, we also employ NRMSE and NMAE to facilitate comparison of estimation performance for different scales as the errors are normalised based on the mean of the actual values [35].

### 2.6. Model Training

Before online (i.e., real-time) usage, machine learning models must be trained offline with available data in order to allow them to learn the relationships between the inputs and the targeted output(s) [68]. Generally, a major limitations of data-driven methods is the need for large datasets that should be employed for training, in order to allow the model to recognise the largest possible occurrences of different patterns. Although our methods are developed to estimate the number of vehicles based on CV data, we propose here to employ for model training high resolution vehicle data collected by different methods and tools. For example, suitable data include vehicle trajectories, such as the ones collected by video cameras, like the Next Generation SIMulation (NGSIM) data [69], or via unmanned aerial vehicles (drones), like the recently collected pNEUMA dataset [70]. Of course, training the models with a large amount of data is desirable, as they would allow to properly identify patterns in the training data that map the input data attributes to the targeted output(s). On the other hand, in order to limit the amount of data that needs to be collected, an alternative approach is to generate synthetic training data, by using a subset of the real data to calibrate a microscopic traffic simulation model, which, in turn, is then used to produce high resolution synthetic data. The latter approach allows to produce massive, virtually unlimited, data needed for training the estimator, while real data may still be used for testing the estimator and assessing its accuracy. After the models are trained, they can be used online for estimation by taking CV data as input.

#### 2.6.1. Data Settings for the Aggregated Estimation Model

**Figure 4:**
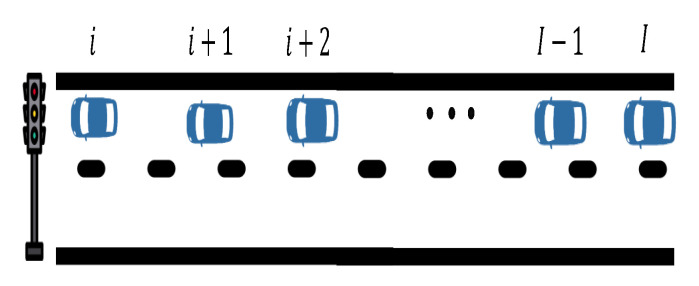
A set of vehicles on a signalised link, which may resemble a frame of video data.

In order to train the aggregated estimation model, a proper dataset that includes input and output variables should be prepared. Let us assume a set of vehicles V(k) approaching a signalised intersection at a given time *k*, as the one shown in Figure 4. Assuming availability of information for all vehicles at each time-step *k*, either obtained from high resolution real data or produced via microscopic simulations, we can tag some vehicles as CVs, considering all the possible subsets $\bar{S}\left(k\right)\subseteq V\left(k\right)$. The total number of possible combinations of CVs is

(19) ∑k=1K∑m=1|V(k)||V(k)|m=∑k=1K∑m=1|V(k)||V(k)|!m!(|V(k)|−m)!.

For each subset of vehicles, we may then calculate aggregated variables according to (3)–(5). Finally, the training dataset at time time *k*, represented in Table 4, is created by including a row for each subset of vehicles, considering the aggregated variables calculated for the vehicles in the subset (i.e., the number of CVs) as inputs and the number of non-connected vehicles, i.e., $\left|V\left(k\right)|-\right|\bar{S}\left(k\right)|$, as output.

#### 2.6.2. Data Settings for the Disaggregated Estimation Model

We describe here the data preparation process for training the disaggregated estimation model, assuming availability of high resolution data that include information such as position, speed, and acceleration for all vehicles in a road section. We assume that the lane where each vehicle is located is available in the collected data. At each time-step *k*, which could be the sampling time of the data, we consider all possible combinations of vehicle pairs and tag the vehicles belonging to each pair as CVs, while vehicles in between are tagged as non-connected. The resulting number of possible combinations is

(20) J=|V(k)|2=|V(k)|!2!(|V(k)|−2)!.

Then, a corresponding row is created for every pair, consisting of actual data for tagged vehicles as inputs and the number of (non-connected) vehicles between them as output. Based on the case illustrated in Figure 4, Table 5 shows an example of such training dataset at time time *k*. For example, in row 2, vehicle *i* and vehicle i+2 are tagged as CVs, while there is one (non-connected) vehicle between them (vehicle i+1); whereas, in row 3, vehicle i+1 and vehicle i+2 are tagged as CVs, while there are zero (non-connected) vehicles between them.

## 3. Data Description

**Figure 5:**
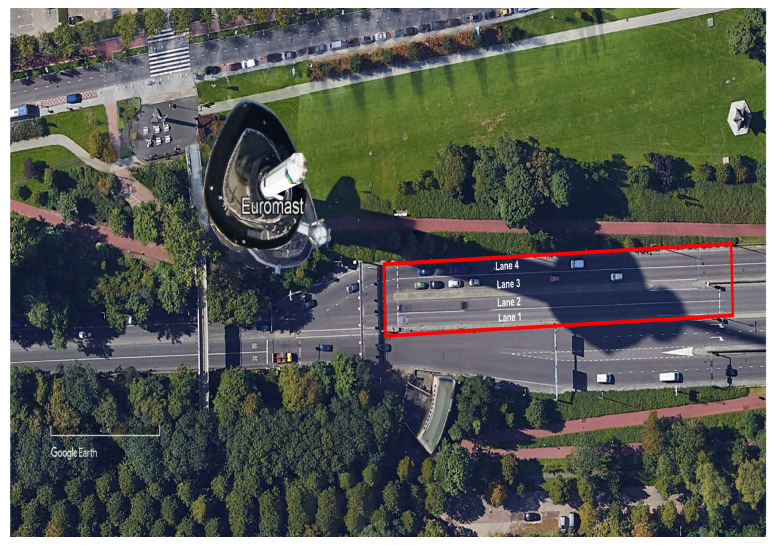
The area of Euromast tower and the signalised segment where data was collected. (Source: Google Earth).

In order to demonstrate the effectiveness of the proposed estimation methods, we employ vehicle data related to a signalised link, collected in Rotterdam, the Netherlands, provided in [71]. The data was collected via high resolution video recording from the top of the Euromast tower: see Figure 5 for an overview of the area. The road segment covered by the video, which starts from the east side of the tower, is 180 m in length, of which about 125 m are located upstream of the traffic light. A traffic signal regulates the traffic flows of two two-lane roads that merge into a two-lane road. The speed limit in the entire stretch is 50 km/h. Data was collected for a total amount of 1.5 h, between 8:30 a.m. and 10:00 a.m. on 18 May 2008; however, only about 80 min of recordings were available for our investigations. Vehicle trajectories are extracted from the videos by considering frames at a frequency of 15 Hz via image processing techniques. The processed dataset include longitudinal position, lane, speed, and acceleration of all vehicles. In total, trajectories of 1168 vehicles are captured, where the share of lane 1, 2, 3, and 4 are 141, 365, 283, and 379 vehicles, respectively. The share of heavy duty vehicles in the traffic flow is 3.5%. As the trajectory of a single vehicle is constructed by processing the images at each timestep, there is possibility of finding many partial trajectories of the same vehicle; in this regard, the dataset was cleaned and extra trajectories for each vehicle were removed. Moreover, since the method used to extract vehicle data from images produced position errors, filtering techniques were applied.

Despite the quality of such data is appropriate for training our models, its size is not deemed sufficient for conducting significant validation experiments. In fact, although there are no strict guidelines about the amount of data required to train satisfactorily an ANN, as it largely depends on the complexity of estimation and the nature of problem, a rule-of-thumb is to have a training dataset that is at least ten times larger than the number of parameters in an ANN network [72]. Accordingly, as the real dataset is limited, sufficient data may not provided in particular for the rare cases such as S-F and S-Q and Q-F. Therefore, we generate synthetic data by using the microscopic traffic simulation software PTV Vissim [73]. We proceed by building a microscopic simulation scenario, where the network has the same road characteristics and traffic signal settings as the use case. A two phase fixed-time signal timing with 60 s cycle time and equal green times for both phases is considered to control the flows of the two merging roads. Traffic demand is specified for each lane based on traffic patterns extracted from the real data. Finally, in order to replicate realistic traffic patterns, we utilise Wiedemann 99 driving behaviour model, using a set of parameters calibrated on the trajectory data in [71,74]. We run simulations for a time horizon of 1 h, which, by collecting second-by-second data, provides a sufficient amount of data to appropriately train the estimation models. Moreover, in Section 4.2, we test different amounts of simulated data, assessing their impact on the estimation performance.

As mentioned in Section 2.4, in order to build our models reducing the risk of overfitting and allowing for unbiased testing, data is split in three different parts, which have different purposes, namely, training, validation, and testing. As training and validation are assumed to be performed offline, for these tasks we use the synthetic data produced via microscopic simulation; in particular, we select data obtained from lanes 1, 2, and 4 for training, while data from lane 3 for validation. This provides sufficient data for training while the models are validated based on data from an independent data source. On the other hand, since testing corresponds to the actual estimation task, which is supposed to happen online, we employ real traffic data, considering data from all four lanes.

In order to cluster data for the disaggregated estimator, as described in Section 2.3.2, we consider for our main experiments $\theta_{1}=5\;\mathrm{km}/h$ and $\theta_{2}=36\;\mathrm{km}/h$. In addition, we perform a sensitivity analysis to investigate the performance of our estimator with different values of $\theta_{1}$ and $\theta_{2}$ in Section 4.4.

The total number of samples for training the models are presented in Table 6. Note that, since the number of data in clusters F-S and F-Q is low, we merge them into a single cluster which is called F-SQ.

## 4. Model Training and Validation

### 4.1. Accuracy of Trained Models

The models are built using Python 3.6 and Tensorflow 2.4.0 [75]. The dropout layers size and $L_{2}$ regularisation parameter are tuned for each individual model based on empirical tuning. A maximum number of 300 epochs is considered for all models.

In order to avoid overfitting, we monitor the errors for the training and validation datasets in each epoch, with the aim of ensuring that the error for the training dataset is not considerably lower than the error for the validation dataset. More detailed information on the checking for overfitting for all the trained models are presented in Appendix A.

Table 7 presents the RMSE and MAE calculated for the training and validation data for all the developed models. A first observation is that all the resulting errors appear small in magnitude; for example, the MAE never exceeds 1 veh for neither training nor validation. Moreover, we observe that the error for the aggregated method are larger than the errors for the disaggregated method, which is due to the fact that the former method uses only aggregated information and delivers as output the total number of non-connected vehicles present in the road stretch; whereas the latter method uses more accurate information and delivers the number of non-connected vehicles between a pair of CVs. For the disaggregated estimation models, better performance is achieved where both vehicles belong to the same cluster, i.e., Q-Q, S-S, and F-F, which can be explained by the fact that it is reasonable to expect that vehicles in between have more homogeneous characteristics (e.g., their space gaps). On the other hand, Q-F is the least accurate model, which is attributed to the fact that there is a large difference in speed (and often position) between the upstream and downstream vehicles, while there is no direct information on, e.g., the queue length. Similarly, in models based on a single CV, model F is the least accurate one.

### 4.2. Impact of Training Dataset Size

**Figure 6:**
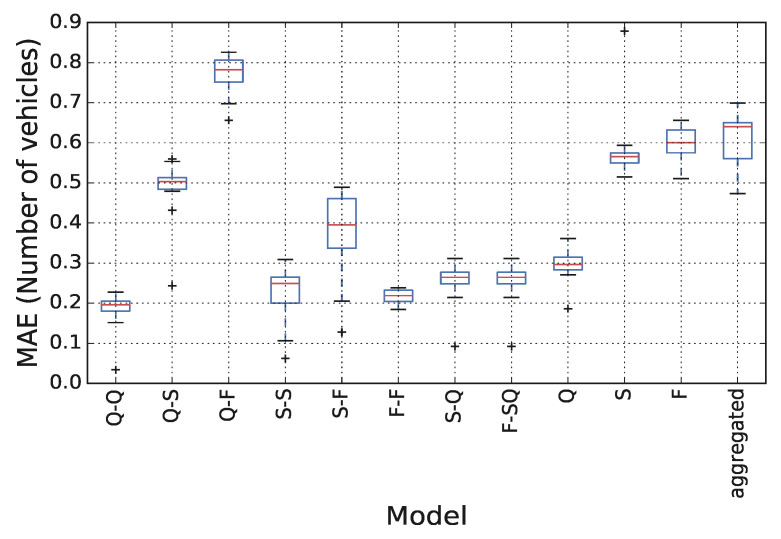
Estimation performance considering different amounts of training data.

As mentioned before, previous results were obtained by training all the models with 1 h of synthetic data. We investigate here the sensitivity of our methods’ accuracy for different amounts of (synthetic) data utilised to train the models. For this purpose, we generate several datasets of synthetic data, each one obtained considering a different simulation horizon, in a range between 15 min and 4 h at 15 min interval. Then, each dataset (corresponding to a specific amount of data) is used to train a full set of models. Figure 6 shows the distribution of MAE for each model, where different amounts of data for model training are considered. We can observe that, for any simulation horizon, the resulting error never exceed 0.9 veh. Moreover, a pattern similar as in Table 7 can be observed, where the average of MAEs for the Q-Q model is the lowest, while the highest average of MAEs is for the Q-F model. Moreover, the MAE range for all models does not exceed 0.4 veh. Therefore, we conclude that, for the tested range of data, the estimation method is slightly sensitive to the amount of data employed for training, which is a promising feature for implementation of these methods, where obtaining large amount of data may be undesirable and challenging. However, one should note that the amount of data available for training and validation is largely different for the different models. For example, models such as S-Q and F-SQ have substantially less data than the other models (e.g., as shown in Table 6, for 1 h of simulated data we have less than 200 samples), which may be too limited for appropriate model training and should be taken into account when defining the training dataset size.

### 4.3. Impact of Clustering on the Accuracy of the Disaggregated Method

**Figure 7:**
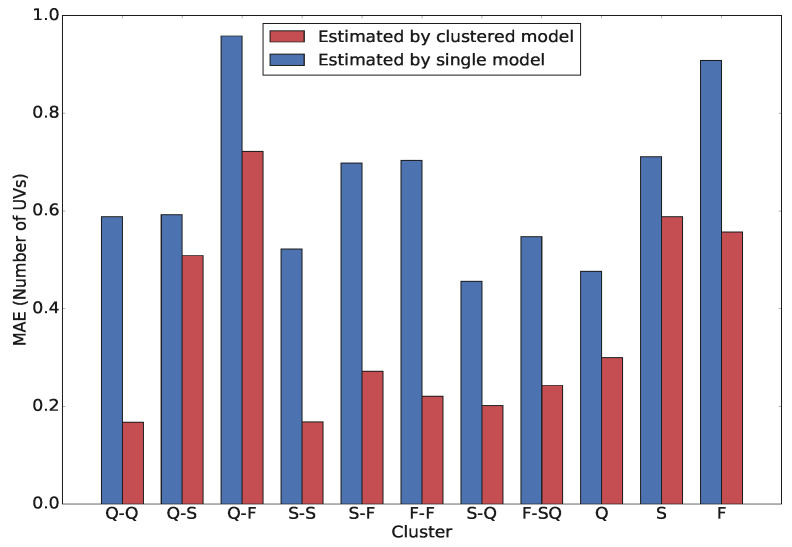
MAE for the cases when a model is built for each cluster and when a unique model is built for all clusters, in the disaggregated approach.

In order to numerically assess and evaluate the implementation of clustering on the models accuracy, we compare the estimation performance for each cluster for both cases when a model is built for each cluster and when a unique model is built for all clusters. Figure 7 illustrates the MAE for these two cases. We clearly observe that using a separate model for each cluster results in more accurate estimation than when a single model is used. The biggest improvement can be seen in clusters where both CVs are in the same phase, i.e, Q-Q, S-S and F-F. For all the cases, both for vehicle pairs and single vehicles, the MAE in the case a single model is applied always exceed 0.5 veh, approaching 1 veh in some cases; on the other hand, while using a model for each cluster, the error never exceeds 0.8 veh, being lower than 0.3 veh in many clusters. This reveals that clustering is highly beneficial in improving estimation accuracy.

### 4.4. Impact of Threshold Values for Clustering

**Figure 8:**
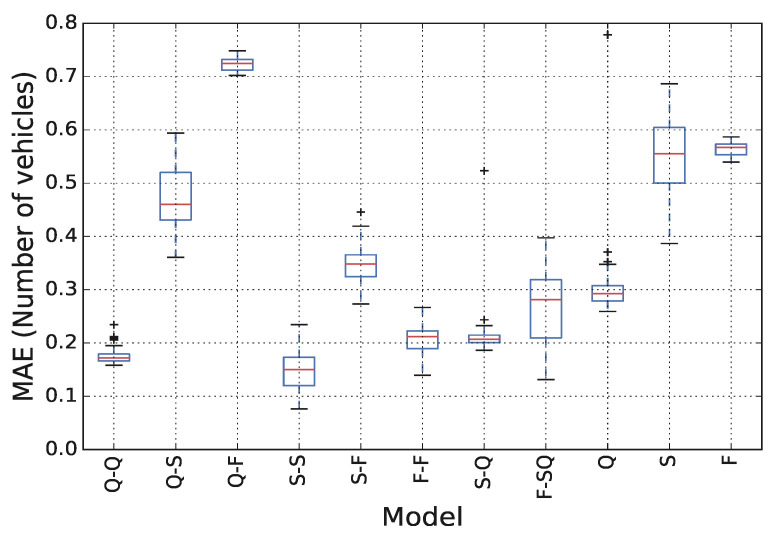
Disaggregated model performance considering different speed thresholds for vehicle phase classification.

We investigate here the impact that different thresholds $\theta_{1}$ and $\theta_{2}$ have on the estimation performance. In particular, we perform a sensitivity analysis considering for $\theta_{1}$ all integer values between 1 km/h and 10 km/h and for $\theta_{2}$ all integer values between 30 km/h and 40 km/h, training all models with all possible combinations of $\theta_{1}$ and $\theta_{2}$. The distribution of MAEs for each model is presented in Figure 8, where we observe that the resulting MAE is not significantly affected by the choice of $\theta_{1}$ and $\theta_{2}$. The highest maximum MAE never exceeds 0.8 veh, while the range of MAEs is less than 0.5 veh for all models. Therefore, we can conclude that, despite $\theta_{1}$ and $\theta_{2}$ alter the composition of different clusters, also in terms of data quantity, their impact on estimation performance is minimal.

## 5. Estimation Performance on Real Data

In this section, we evaluate the performance of the trained models on the real data. Firstly, we focus on the disaggregated method, reporting results in term of estimation of the number of vehicles between a pair of CVs. Then, we turn our attention on the aggregated model, assessing it capabilities in in estimating the total number of vehicles employing CV data.

### 5.1. Estimation of Number of Vehicles between a Pair of CV

Here, we evaluate in more details the performance of the disaggregated models. RMSE and MAE obtained while implementing the estimator on real data are presented in Table 8. Except Model F and S, the resulting RMSE is equal or less than 1 veh, while the resulting MAE for all models never reaches 0.8 veh. Qualitatively, we see a similar pattern as the one observed in model training using synthetic data (e.g., Model Q-Q has the lowest RMSE and MAE); however, the errors calculated with real data are slightly higher. This could be explained by the fact that synthetic (simulated) data is more consistent than real data, since it is generated based on mathematical driving behaviour models, which, despite incorporating some stochastic components, feature more predictable characteristics. On the other hand, the real data features the heterogeneity, perception inaccuracies, and unpredictable situations that actually appear in human driving.

**Figure 9:**
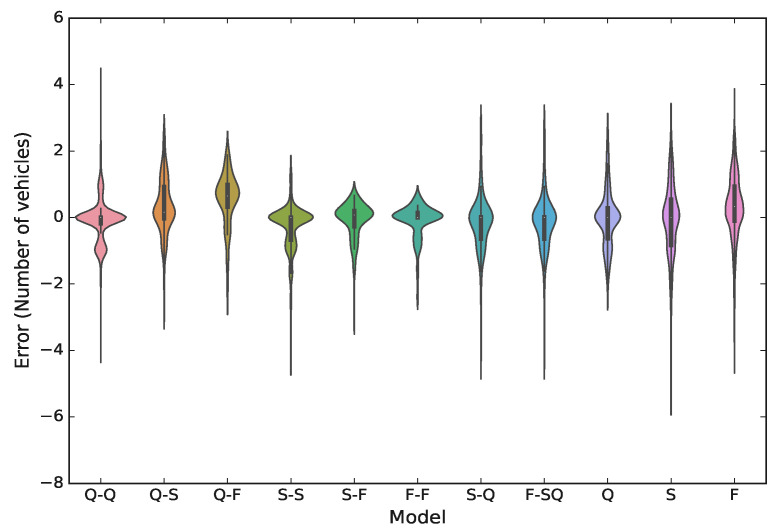
Estimation error distribution obtained for real data.

In Figure 9, we show violin plots for the testing error of all models on real-world data, defining the error $E_{i}$ of the *i*-th estimation as

(21) $$E_{i}=\hat{y_{i}}-y_{i},$$

where $\hat{y_{i}}$ is the estimate of $y_{i}$. By inspecting disaggregated models results, we observe the same pattern as for the training phase; for example, when both vehicles are in the same cluster, i.e., Q-Q, S-S, and F-F, the error ranges are considerably smaller. In contrast, the highest error range can be seen for the Q-F model, where the vehicles are from different clusters, which, as before, can be explained by the fact that there is considerable gap between speed of CVs in these models. In other words, our method produces better estimates when the traffic characteristics are more homogeneous, which is reasonably expected. In models that have been trained to estimate the number of non-connected vehicles between a CV and the stop-bar, the best performance is achieved for the Q model, where the error is close to zero; whereas, the highest level of uncertainty can be seen in the F model.

**Figure 10:**
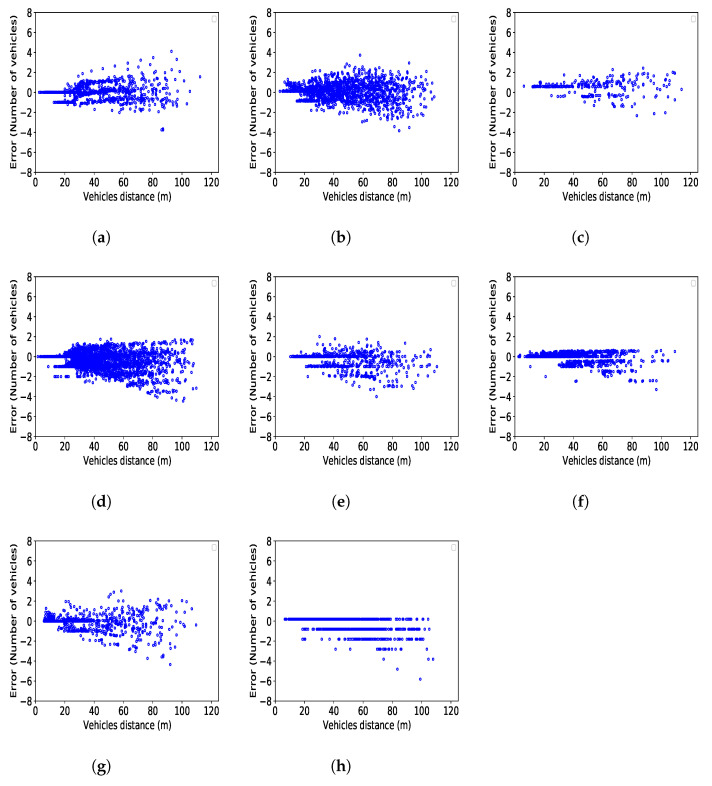
Error of estimations based on distance of CV pairs. (**a**) Q-Q model; (**b**) Q-S model; (**c**) Q-F model; (**d**) S-S model; (**e**) S-F model; (**f**) F-F model; (**g**) S-Q model; (**h**) F-SQ model.

According to the Pearson correlation coefficient [76], estimation errors are highly correlated with the distance between vehicles in a pair. To further investigate this, we show in Figure 10 the influence that the distance between CVs has on the estimation error. In most cases, as the distance between CVs increases, we observe that the magnitude of the error also increases. Moreover, for almost all models, in short distances, the estimation is extremely accurate. For instance, in the Q-Q model, we obtain a perfect estimate when the distance is shorter than 10 m, while, when the distance is between 10 m and 20 m, the error never exceeds 1 veh. Moreover, we can see that the maximum error is never higher than 4 veh, with very few exceptions, whereas the majority of errors in all models are between −1 and 1 veh. To summarise, we can state that, for all models, estimates based on pairs where CV are at a smaller distance are the most reliable, while the level of uncertainty increases as the distance between CVs increases. Finally, inspecting Figure 9 and Figure 10, we can see that the only cases when high errors (e.g., 5 veh) appear are when the distance between two CVs is very high, e.g., more than 80 m. In this situation, identifying a relation from CVs data and the number of vehicles between such distant CVs is indeed not an easy task, but, still, the resulting error is not extremely high. Note also that this situation is expected to be seen only in case the amount of CVs is very low.

### 5.2. Evaluation of Vehicle Counting Estimation

The total number of vehicles approaching a signalised segment can be estimated using both the aggregated and disaggregated methods, via (7) and (11), respectively. Here, we compare the two estimators, considering the number of CVs and their penetration rates separately.

**Figure 11:**
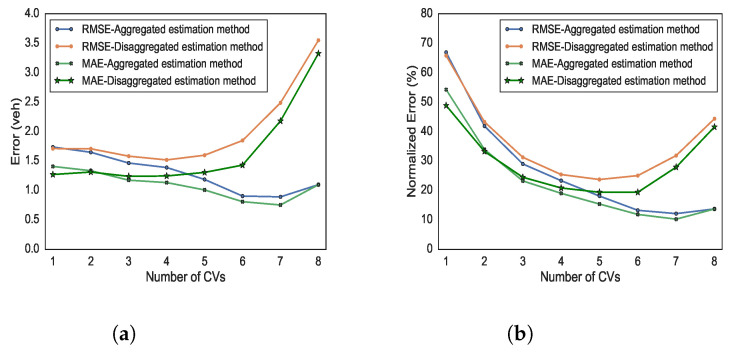
Accuracy measure of estimated total vehicle counts based on the number of CVs (**a**) RMSE and MAE (**b**) NRMSE and NMAE.

Figure 11 shows a comparison in term of estimation error of the total vehicle counts for both the aggregated and disaggregated methods; RMSE and MAE are shown in Figure 11a, while and NRMSE and NMAE are shown in Figure 11b. We can observe that the aggregated estimation method consistently outperforms the disaggregated estimation method, for any number of CVs present within the stretch. Moreover, as the number of CVs increases, the performance of the disaggregated estimator deteriorates. The main reason is that, by increasing the number of CVs, the number of “pair” estimations, included in the first term of (11), increases. Therefore, the error characterising each separate estimate accumulates, producing a higher total error. On the other hand, RMSE and MAE calculated for the aggregated estimation method decrease as the number of CVs increases. In particular, when the number of CVs is higher than six, RMSE and MAE are less than 1 veh, indicating a very good estimation performance; moreover, we see that the two metrics converge, implying that the absolute values of the errors are similar for all data points. Normalised measures show a similar behaviour except that NRMSE and NMAE for the disaggregated estimator decreases at first when the number of CVs is increasing, until we reach five CVs; then, the NRMSE of the disaggregated estimator increases, exceeding 40% for eight CVs (the maximum number witnessed in our experiments). In contrast, the aggregated estimator improves its accuracy as the number of CVs increases, reaching a minimum where NRMSE is lower than 10%. Note that, a reason why we observe very high percentage errors in the low number of vehicle is that even a small absolute error may lead to high relative error. For instance, if the actual number of vehicles is one but the model estimates two vehicles, then the relative error is 100%.

**Figure 12:**
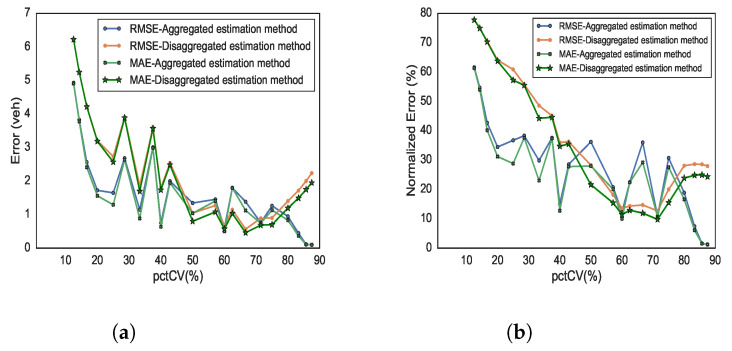
Accuracy measure of estimated total vehicle counts based on the pctCV of CVs (**a**) RMSE and MAE (**b**) NRMSE and NMAE.

Figure 12a,b present performance metrics for the absolute and normalised estimation errors, respectively. Inspecting the error trend reveals that, at low and moderate pctCV, both estimators have similar performances, with an improvement in estimation quality as the pctCV increases up to about 70%. However, in the case of higher pctCV, we see that the aggregated estimator performance improves, while the disaggregated estimator performance deteriorates. Note that the unsmooth plots are due to the fact that the pctCV value is dependent (and sensitive) to the number of CVs and the total number of vehicles. For example, 50% pctCV can be achieved with 4 CVs and 8 vehicles in total or with 1 CVs and 2 vehicles in total, whereas some pctCV can only be achieved with a specific combination of CVs/total number of vehicles.

**Figure 13:**
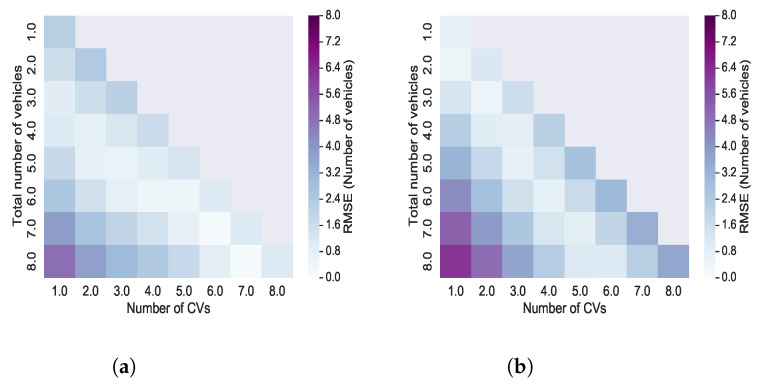
RMSE of estimation as function of the total number of vehicles and the number of CVs (**a**) Aggregated estimator (**b**) Disaggregated estimator.

Finally, we present in Figure 13a heat map that shows the RMSE for the aggregated and disaggregated estimators as a function of both the number of CVs and the total number of vehicles. One may observe that, for the both estimators, the highest error (i.e., the darkest cell) occur when information from only one CV is used to estimated eight vehicles. A similar pattern can be seen for both estimators unless number of CVs is close to number of total vehicles. In this case, the disaggregated estimator accuracy decreases while the aggregated estimators has accurate estimations in the similar condition.

## 6. Discussion and Conclusions

In this paper, we proposed data-driven methods to estimate the total number of vehicles at a signalised intersection using only data collected from a limited amount of CVs. In particular, we propose and test two estimation methods: an aggregated method, which employs aggregated data from CVs and delivers the total number of vehicles on a signalised urban link; and a disaggregated method, which employs data from a pair of CVs to estimate the number of non-connected vehicles between that CV pair. Both methods satisfy the initial objectives set for their design. We have seen that using clustering for building different models in the disaggregated method leads to an improvement in term of estimation accuracy, consistently outperforming the case when a unique model was utilised. Moreover, our results show that the error obtained for all models in the disaggregated method is largely affected by the distance between CVs in a pair. Additionally, a more accurate estimation is achieved when the speed difference within a CV pair is lower. We also observed that, in case the results obtained via the disaggregated method are used to estimate the total number of vehicles, estimation is worse than when simply using the aggregated method. This is attributed error accumulation, which is indeed more pronounced in the case of high pctCV. On the other hand, the aggregated method features better performance as the pctCV increases. This suggests that, in case both the total number of vehicles and more granular estimations are needed, it is wiser to implement a combination of the proposed methods.

This research is one of the first data-driven efforts in estimating vehicle counting, relying only on CV data. The findings of this paper show that data-driven methods, based, e.g., on machine learning models, can produce useful results in estimating traffic variables during the transition period until we reach a fully connected environment. A main practical implication of the proposed method is that it essentially allows vehicle counting needless of infrastructure-based sensors, such as loop detector, even with a low amount of CVs. In addition, we demonstrate that even a low amount of real data may be successfully employed for training the proposed models by using a synthetic dataset generated by a calibrated simulation tool.

In future work, we aim at investigating the usage of filtering methods to improve estimation accuracy, by utilising time-series data in addition to instantaneous measurements. Moreover, developing a combination of both aggregated and disaggregated approaches, exploiting the strengths and mitigating the weaknesses of both methods, to estimate with higher accuracy the position of non-connected vehicles is an interesting future research direction. Finally, it may be worth investigating how the accuracy achieved in calibrating the simulation tool affects the estimation accuracy, as well as testing other simulation tools.

## Figures and Tables

**Table 1:** Summary of research on traffic state estimation using CV data.

Research Work	Estimated Quantities	Spatial Resolution	Time Resolution	Utilised Data	Estimation (Main) Method	Validation Data
Ramezani et al. [18]	queue profile	link	signal cycle	only CV data	shockwave analysis; data mining	real data
Zheng et al. [29]	traffic volumes	lane	10 min–1 h	vehicle trajectories and signal status	maximum likelihood	real data
Zhao et al. [30]	queue length; traffic volume	link	1 h	only CV data	probability theory	real and simulated data
Ramezani et al. [18]	queue profile	link	signal cycle	only CV data	shockwave analysis; data mining	real data
Gao et al. [42]	queue length	lane	signal cycle	only CV data	shockwave sensing and neural network	simulated data
Aljamal et al. [31,32,41,43,44]	traffic density	lane	variable	CV and detector data	combination of ANN and RF, K-NN, Kalman filter, adaptive kalman filter, and non-linear Particle filter	real and simulated data
Nguyen Van Phu et al. [24]	penetration rates; vehicles arrival rate; turning ratios; queue lengths	lane	second	only CV data	probability theory	simulated data
Proposed method	total number of vehicles; number of vehicles upstream and downstream of each CV	lane and intra-lane	second	only CV data	machine learning (ANN)	real data

**Table 2:** Criteria for clustering CV pairs in the disaggregated model.

Model	Traffic Phase of Downstream CV	Traffic Phase of Upstream CV	Speed of Downstream CV	Speed of Upstream CV
Q-Q	queue	queue	$v_{i}<\theta_{1}$	$v_{i+1}<\theta_{1}$
Q-S	queue	slowing-down	$v_{i}<\theta_{1}$	$\theta_{1}\leq v_{i+1}\leq \theta_{2}$
Q-F	queue	free-flow	$v_{i}<\theta_{1}$	$v_{i+1}\geq \theta_{2}$
S-S	slowing-down	slowing-down	$\theta_{1}\leq v_{i}\leq \theta_{2}$	$\theta_{1}\leq v_{i+1}\leq \theta_{2}$
S-F	slowing-down	free-flow	$\theta_{1}\leq v_{i}\leq \theta_{2}$	$v_{i+1}\geq \theta_{2}$
F-F	free-flow	free-flow	$v_{i}\geq \theta_{2}$	$v_{i+1}\geq \theta_{2}$
S-Q	slowing-down	queue	$\theta_{1}\leq v_{i}\leq \theta_{2}$	$v_{i+1}<\theta_{1}$
F-Q	free-flow	queue	$v_{i}\geq \theta_{2}$	$v_{i+1}<\theta_{1}$
F-S	free-flow	slowing-down	$v_{i}\geq \theta_{2}$	$\theta_{1}\leq v_{i+1}\leq \theta_{2}$

**Table 3:** Criteria for clustering the CVs closest to the stop-bar in the disaggregated model.

Model	Traffic Phase of CV	CV Speed
Q	queue	$v_{i}<\theta_{1}$
S	slowing-down	$\theta_{1}\leq v_{i}\leq \theta_{2}$
F	free-flow	$v_{i}\geq \theta_{2}$

**Table 4:** Data structure defined for the aggregated model.

*m*	Set of Vehicles	Aggregated Variables for Each Vehicle Set *	|S|	|V|−|S|
1	{*i*}	...	1	I−i
1	{i+1}	...	1	I−i
1	{i+2}	...	1	I−i
...	...	...	...	...
1	{I}	...	1	I−i
2	{i,i+1}	...	2	I−i−1
2	{i,i+2}	...	2	I−i−1
...	...	...	...	...
3	{i,i+1,i+2}	...	3	I−i−2
...	...	...	...	...
|V|	{i,i+1,i+2,...,I}	...	I−i+1	I−i−J−1

* For the sake of space, we do not show aggregated variables for each set of vehicles in this table. Details can be found in Section 2.2.

**Table 5:** Data structure defined for the disaggregated model.

	$d_{d}$	$d_{u}$	$d_{d}-d_{u}$	$v_{d}$	$v_{u}$	$v_{d}-v_{u}$	$a_{d}$	$a_{u}$	$a_{d}-a_{u}$	$u_{d}$	$u_{u}$	$T_{d}$	$T_{u}$	$n_{d,u}$
1	$d_{i}$	$d_{i+1}$	$d_{i}-d_{i+1}$	$v_{i}$	$v_{i+1}$	$v_{i}-v_{i+1}$	$a_{i}$	$a_{i+1}$	$a_{i}-a_{i+1}$	$u_{i}$	$u_{i+1}$	$T_{i}$	$T_{i+1}$	0
2	$d_{i}$	$d_{i+2}$	$d_{i}-d_{i+2}$	$v_{i}$	$v_{i+2}$	$v_{i}-v_{i+2}$	$a_{i}$	$a_{i+2}$	$a_{i}-a_{i+2}$	$u_{i}$	$u_{i+2}$	$T_{i}$	$T_{i+2}$	1
3	$d_{i+1}$	$d_{i+2}$	$d_{i+1}-d_{i+2}$	$v_{i+1}$	$v_{i+2}$	$v_{i+1}-v_{i+2}$	$a_{i+1}$	$a_{i+2}$	$a_{i+1}-a_{i+2}$	$u_{i+1}$	$u_{i+2}$	$T_{i+1}$	$T_{i+2}$	0
...	...	...	...	...	...	...	...	...	...	...	...	...	...	...
*J*	$d_{I-1}$	$d_{I}$	$d_{I-1}-d_{I}$	$v_{I-1}$	$v_{I}$	$v_{I-1}-v_{I}$	$a_{I-1}$	$a_{I}$	$a_{I-1}-a_{I}$	$u_{I-1}$	$u_{I}$	$T_{I-1}$	$T_{I}$	0

**Table 6:** Training, validation, and testing datasets size for each model.

Model	Training Dataset Size (Synthetic Data)	Validation Dataset Size (Synthetic Data)	Testing Dataset Size (Real Data)
Aggregated	137,370	34,343	369,799
Q-Q	2706	810	1461
Q-S	1064	313	1881
Q-F	1267	359	293
S-S	419	73	4479
S-F	492	145	652
F-F	1255	356	1376
S-Q	171	43	763
F-SQ	173	54	987
Q	3750	1270	3723
S	1854	580	6956
F	4996	1702	4175

**Table 7:** Performance measures for the proposed estimation methods.

Model	RMSE (veh)		MAE (veh)	
	Training	Validation	Training	Validation
Aggregated	0.7673	0.8150	0.5415	0.6464
Q-Q	0.3333	0.3678	0.1675	0.2031
Q-S	0.6517	0.6959	0.5087	0.5452
Q-F	0.8803	0.8967	0.7218	0.7838
S-S	0.3646	0.3169	0.1682	0.1430
S-F	0.4541	0.5467	0.2716	0.3121
F-F	0.3564	0.3302	0.2204	0.2205
S-Q	0.3538	0.4452	0.2015	0.2501
F-SQ	0.3737	0.3574	0.2428	0.2500
Q	0.4988	0.4730	0.2999	0.3133
S	0.8491	0.9520	0.5883	0.6774
F	0.7944	0.8030	0.5567	0.5848

**Table 8:** Performance measures for the disaggregated estimation methods on real data.

Model	RMSE (veh)	MAE (veh)
Q-Q	0.6627	0.3956
Q-S	0.8892	0.6634
Q-F	0.8901	0.7810
S-S	0.8103	0.4732
S-F	0.9698	0.6401
F-F	0.5130	0.3282
S-Q	0.8841	0.5477
F-SQ	0.8358	0.5368
Q	0.9696	0.6700
S	1.1023	0.7670
F	1.0010	0.7628

**Figure A1:**
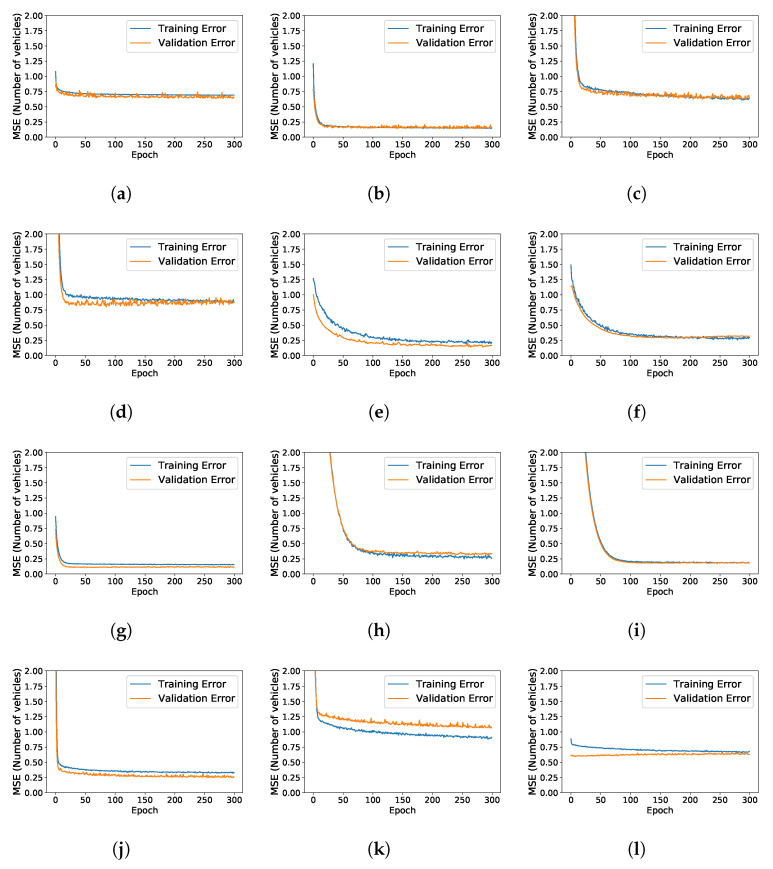
Training and validation results for all the built models. (**a**) Aggregated Model; (**b**) Q-Q model; (**c**) Q-S model; (**d**) Q-F model; (**e**) S-S model; (**f**) S-F model; (**g**) F-F model model; (**h**) S-Q model; (**i**) F-SQ Model; (**j**) Q model; (**k**) S model; (**l**) F model.

## Data Availability

Not applicable.

## Appendix A

We present here some results related to the training and validation of the proposed models. Figure A1 shows a comparison of errors for the training and validation datasets for all trained model. We observe that the MSE is decreasing while the training process is going forward in both the training and validation dataset. Moreover, the MSE for the validation dataset is never higher than MSE for the training dataset, which indicates an absence of overfitting during the training.
